# Preparation and application of nanostructured carbon from oil fly ash for growth promotion and improvement of agricultural crops with different doses

**DOI:** 10.1038/s41598-022-21639-w

**Published:** 2022-10-11

**Authors:** Saleh M. Alluqmani, Nadiyah M. Alabdallah

**Affiliations:** 1 Department of Physics, Faculty of Applied Science, Umm Al-Qura University, Makkah, 21955 Saudi Arabia; 2 Department of Biology, College of Science, Imam Abdulrahman Bin Faisal University, P.O. Box 1982, Dammam, 31441 Saudi Arabia

**Keywords:** Biological techniques, Physiology, Plant sciences, Materials science, Nanoscience and technology, Physics

## Abstract

Application of carbon nanomaterials (CNMs) in agricultural production has piqued the interest of researchers. However, despite the enormous importance of CNMs in plant development, little is known about the effects of carbon nanoparticle (CNP) doses on plant physiological responses. Therefore, the aim of the current study was to check the effects of nanostructured carbon derived from oil fly ash (COFA), which was derived for the first time from high-energy ball-milling followed by a sonication process, on *Phaseolus vulgaris* L. and *Cicer arietinum* L. plants. We evaluated the plant physiological and biochemical parameters of the COFA-treated seedlings. Two different doses (4 mg L^−1^ and 8 mg L^−1^) of COFA and a control were studied. The results indicated that the germination rate (%), shoot length, root length, pod length, leaf area, fresh weight and dry weight were increased with the addition of COFA. Likewise, COFA increased the contents of chlorophyll pigments (Chla, Chlb, carotenoids), proteins, and carbohydrates in both species compared to the control. Finally, these findings showed that a COFA treatment at 4 mg L^−1^ after ball milled-sonication in water (BMW4) constituted the best dose for growth and physiology. Our findings reveal that the novel strategy of COFA engineering led to a boost in the growth of *Phaseolus vulgaris* and *Cicer arietinum*. Our results have high potential for agricultural research and provide an impact on food security.

## Introduction

Nanotechnology involves engineering and manipulation of materials on a nanometre scale (1 to 100 nm) to generate biomaterials, sensors, and advanced technologies^1–3^. Carbon-based nanomaterials (CNMs) exhibit unusual physicochemical features at this size, along with a normal surface shapes and a high surface areas. CNMs such as fullerene (C60) are widely used in electronics and biomedicine^3^, while carbon nanotubes (CNTs) are commonly used as drug delivery carriers and in computers^4^. Moreover, reduced graphene oxide is frequently employed in lithium-ion batteries as well as for pollutant remediation^5^. CNMs will unavoidably be discharged into the environment throughout their life cycles since these applications are expanding^6^. As a result, better comprehension of the environmental consequences of CNMs is critical.

Carbon nanoparticles (CNPs) released into the terrestrial ecosystem by eutrophication, agronomic usage, or other mechanisms persist in the soil in high quantities due to the limited soil mobilities of CNPs^7,8^. Plants are the foundation of all ecosystems, and they play critical roles in distribution and transport of CNPs in the ecosystem through uptake and bioaccumulation along the food chain^9^. The absorption capacities of CNPs are substantial due to their large surface areas, and as a result, they can make significant contributions to pollution disposal and soil remediation. CNP_S_ have favourable effects on plant growth and physiology by enhancing plant growth, photosynthetic pigment content, and protein content. To counteract the detrimental effects of CNPs, nonenzymatic and enzymatic antioxidants, as well as a range of other compounds, are effective in protecting plants against the damaging effects of free radicals^10^.

The shaped particle sizes and the structures of carbon nanostructures impact their applicability^11^. For developed nanomaterials, high-energy ball milling has proven to be an effective method for manufacturing nanocrystalline powders^12^. Recently, many studies have reported successful realization of carbon nanostructures engineered from oil fly ash waste by high-energy ball-milling techniques, with sizes ranging from 100 to 20 nm for agglomerated clusters; these provide highly graphitic compositions, large specific surface areas, porosity, nontoxicity, strength, and scattering responses, all of which are promising results^13–16^. Moreover, Numan et al. successfully used a sonication method to reduce the sizes of oil fly ash particles to a few nanometres^13^. However, ball-milled oil ash seems to be finer in shape than sonicated nanoparticles. In our recent study, we used oil fly ash as a starting material for production of powdered carbon nanoparticles with sizes less than 100 nm by high-energy ball milling^16^. This novel route was aimed at removing solid oil fly ash waste and using it as a successful dopant for titanium oxide films. However, these new studies could provide possible agricultural applications. High-energy ball-milling followed by sonication is a novel strategy for enhancing morphological properties. Thus, the technology might offer unique possibilities for oil fly ash engineering, allowing for eco-friendly and sustainable manufacturing from industrial waste. Therefore, exploitation of oil fly ash and manufactured carbon nanostructures could provide agricultural applications that should be checked for the first time.

*Phaseolus vulgaris* L. and *Cicer arietinum* L. are economically significant crops grown around the globe^17,18^. Due to their ability to respond to several environmental conditions, *Phaseolus vulgaris* L. and *Cicer arietinum* L. were chosen as the experimental crops with which to study the effects of carbon nanostructures in oil fly ash doses. We aimed to examine the relationships between crops and COFA samples prepared by high-energy ball-milling and ball-milled samples followed by a sonication process by using physiological and morphological characteristics. Various criteria have been chosen to determine the influence of accumulated COFA materials on morphological and physiological parameters such as chlorophyll pigments and plant growth. Thus, the present study is needed because (i) it proposes a new strategy for preparing synthetic carbon nanoparticles from oil fly ash and (ii) it aids in identifying the maximum permitted concentrations of prepared carbon nanoparticles in crops, which will enable development of novel biotechnological methods for crop development.

## Materials and methods

### Synthesis and characterization of COFA samples

**Figure 1:**
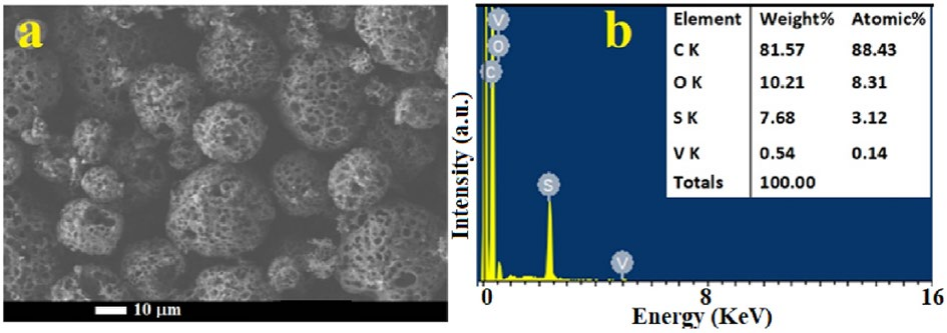
(**a**) SEM images and (**b**) energy dispersive spectroscopy analyses of the waste solid from oil fly ash^15^.

**Figure 2:**
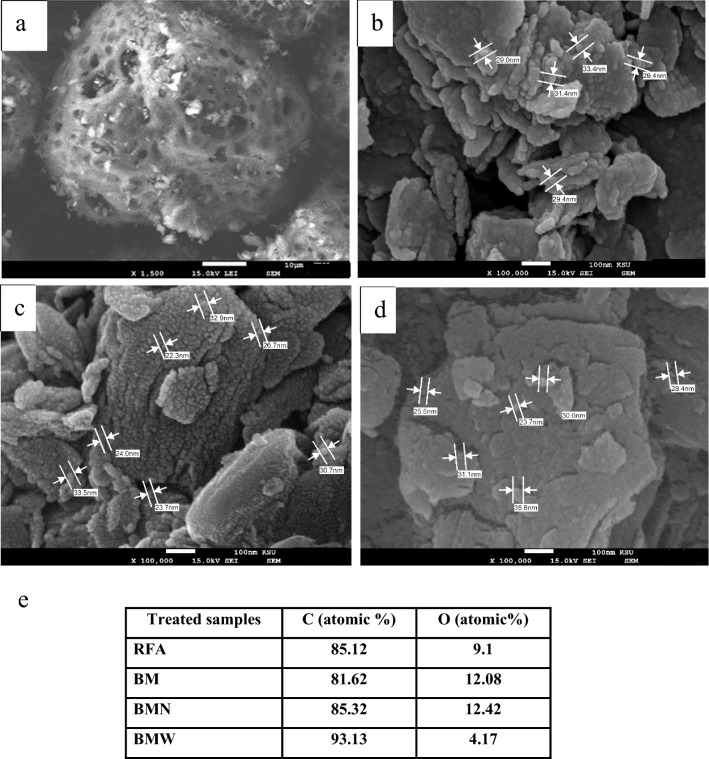
SEM images of (**a**) RFA, (**b**) BM, (**c**) BMN, (**d**) BMW and (**e**) EDS elemental analyses.

**Figure 3:**
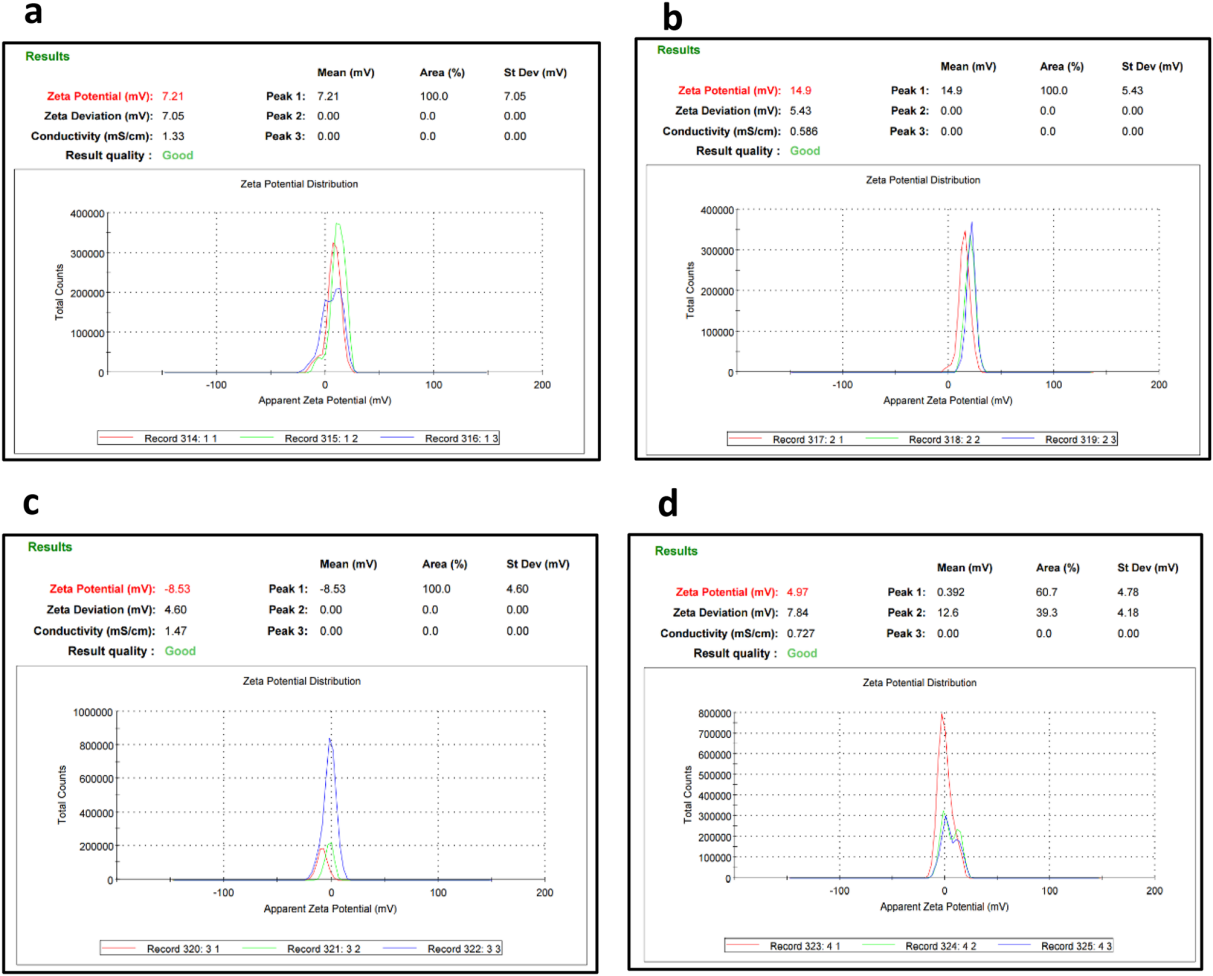
Zeta-potential measurements of (**a**) RFA, (**b**) BM, (**c**) BMN, and (**d**) BMW.

A water desalination station in Jeddah city, Saudi Arabia, provided the raw sample of oil fly ash material. The high-energy ball-milling method was applied to produce powdered carbon nanoparticles from oil fly ash in a dry medium, as reported previously^13^. The elemental composition of the raw material, as determined in our previous report^15^, consisted of 88.43% unburned carbon (C), 8.31% oxygen (O), 3.12% sulfur (S) and 0.14% vanadium (V), as shown in Fig. 1. In a further step, the milled powder was sonicated in liquid media. A total of 10 g of this fly ash was kept for 45 h in a 250 mL agate-based jar. The balls used were made of exceptionally pure agate, and they weighed approximately 11 g and measured 15 mm in diameter. The raw fly ash labelled as RFA was milled to the nanoscale at 400 rpm in an ambient atmosphere using a horizontal oscillatory mill (PM 400; Retsch, Hann, Germany) set to 25 Hz, which produced ball milled powder labelled as BM. The samples were washed, filtered and slowly dried at 50 °C. BM samples were sonicated in deionized water and labelled BMW, or nitric acid (HNO_3_) solution (2 mol L^−1^) and labelled BMN. Then, the BMW and BMN samples were slowly dried. Finally, the morphologies and elemental compositions of the engineered samples were revealed by using scanning electron microscopy (SEM) and energy-dispersive X-ray spectroscopy (EDS) analyses with a JEOL-7600F SEM instrument (Japan). SEM images are shown in Fig. 2. The morphological features of the prepared samples were enhanced (from a to d). It is noticeable that the nanopowder showed an increase in the abundance of clustered micropore structures but offered a high surface area and nano-sized particles (less than 35 nm) influenced by the type of treatment used in this study. Moreover, these features could provide unique access to several applications in the future^11,19^. Figure 2e shows the EDS chemical compositions of the prepared samples, which confirmed the effects of the synthetic process on the carbon and oxygen ratios. Additionally, Fig. 3 presents the results of the zeta potential analysis of synthesised samples. The zeta potential values of RFA, BM, BMN and BMW samples were at 7.21, 14.9, − 8.53 and 4.97 mV, respectively, which indicate the large cluster sizes as a result of lack of electrostatic repulsion.

### Growth conditions

As plant materials, both *Phaseolus vulgaris* cv. Nebraska and Cicer arietinum cv. Bittle-98 were chosen for the present study, which was carried out at the Department of Biology, College of Science, Imam Abdulrahman Bin Faisal University (26.3928° N, 50.1926° E). *P. vulgaris* and *C. arietinum* seeds were obtained from the Altuwaijri company in Saudi Arabia. The strawberry seeds were sterilized with 4% sodium hypochlorite. The experiment used a split plot arrangement with a completely randomized design. Compost, sand (44.2%), silt (35.3%), and clay (23.2%) were used to fill pots (40 cm in height and 25 cm in diameter). In each of these pots, seedlings of *P. vulgaris* and *C. arietinum* were sown. Growing seedlings were watered twice weekly prior to application of experimental treatments. All of the experiments in this investigation were carried out in accordance with applicable institutional, national, and international guidelines and legislation.

### COFA treatment

COFAs were suspended in doubly-distilled water to obtain 4 and 8 mg L^−1^ concentrations and homogenized by sonication for 30 min before use. Several COFA treatments were used in the present experiments, such as the control, ball milled-BM4 (4 mg L^−1^) and BM8 (8 mg L^−1^), raw fly ash-RFA4 (4 mg L^−1^) and RFA8 (8 mg L^−1^), ball milled-sonication in nitric oxide, BMN4 (4 mg L^−1^) and BMN8 (8 mg L^−1^), and ball milled-sonication in water BMW4 (4 mg L^−1^) and BMW8 (8 mg L^−1^).

### Germination percentage

The germination percentage was measured according to the methods in Ref.^20^. The following formula was used: GP = 100 (NG/NT), where NG represents germinated seeds and NT indicates total seeds.

### Determination of growth parameters

The lengths of shoots and roots were measured on a metric scale and expressed in centimetres (cm). Pod lengths were measured by using a micrometer screw gauge. To eliminate sand particles from the plant materials, doubly-distilled water was used to clean them. An analytical balance (HR-60) was used to measure the fresh and dry weights (FW and DW), which are presented in grams (g). On the other hand, an LI-3000C portable leaf area metre was employed for measuring leaf areas.

### Determinations of photosynthetic pigments

The contents of chlorophyll or photosynthetic pigments were determined based on the methods^21^. A 0.25 g leaf sample was collected from each species and milled with 5 mL acetone (80%). The derived plant extract was collected and centrifuged for 15 min at 400 °C and 3000 rpm. Finally, the contents of chlorophyll pigments (Chla and Chlb) were calculated by measuring the absorbances at 663 and 645 nm.

### Determination of protein content

Bradford's technique was used to calculate the total amount of soluble proteins. Four millilitres of cold sodium phosphate buffer (pH 7.4) was dissolved with 0.1 g of leaf tissue, centrifuged, and adjusted to a final volume of 10 mL^22^. To 0.1 mL of extract, 0.5 mL of diluted Bradford Reagent (1:5) was added to achieve the desired result. A spectrophotometer was used to measure the absorbance at 595 nm after 30 min of incubation at room temperature. A standard curve for bovine serum albumin (BSA) was used to measure the protein content.

### Carbohydrate content measurements

Total carbohydrates were determined in accordance with the method of Herbert et al.^23^. A known mass of dry tissue (0.2–0.5 g) was added to 10 mL of sulfuric acid (1 N). The tube was closed and dried in an oven at 100 °C. Colorimetric determinations of total sugars were carried out in accordance with the method of Smith et al. (1956). To prepare the solution, 1 mL of sugar solution was mixed with 1 mL of 10% aqueous phenol solution, and then added to 5.0 mL of concentrated sulfuric acid until well mixed. After they were fully shaken, the tubes were washed with tap water at 23–30 °C and dried for 20 min. A Shimadzu spectrophotometer was used to determine the optical density of the generated colour at a wavelength of 490 nm.

### Statistical analyses

MINITAB-17 statistical software was used to perform the analysis of variance (ANOVA), and the findings are presented as the treatment mean ± SE (n = 3). The LSD test indicates that bars with the same letter do not differ substantially at the *p* ≤ 0.05 level.

## Results

COFA had significant effects on the germination percentages of both *P. vulgaris* and *C. arietinum*. The maximum percentage of germination was observed when carbon nanoparticles were applied to both species at BMW4 (4 mg L^−1^). We studied a variety of morphological traits, including shoot length, root length, pod length, leaf area, fresh weight and dry weight, to assess the favourable impacts of COFA on *P. vulgaris* and *C. arietinum* species. Shoot lengths, root lengths, pod lengths, leaf areas, and fresh and dry root weights were significantly increased (*p* ≤ 0.001) when *P. vulgaris* and *C. arietinum* were subjected to different COFA treatments (BM4, BM8, RFA4, RFA8, BMN4, BMN8, BMW4, and BMW8), as shown by comparison to the control (Tables 1, 2).

**Table 1:** Impact of CNP treatments on the growth parameters of *Phaseolus vulgaris* seedlings.

Growth parameters	Treatment								
	Control	Ball milled (BM)		Raw fly ash (RFA)		Ball milled-sonicated in nitric oxide (BMN)		Ball milled-sonicated in water (BMW)	
		BM4 (BM4 mg L^−1^)	BM8 (BM8 mg L^−1^)	RFA4 (RFA4 mg L^−1^)	RFA8 (8 mg L^−1^)	BMN4 (4 mg L^−1^)	BMN8 (8 mg L^−1^)	BMW4(4 mg L^−1^	BMW8(8 mg L^−1^)
Germination (%)	70.5 ± 5.8h	82.6 ± 6.3f	80.5 ± 6.2g	88.9 ± 6.4e	90.23 ± 7.3d	97.34 ± 7.8b	95.5 ± 8.3c	99.3 ± 8.8a	99 ± 8.6a
Shoot length	55.12 ± 4.1i	60.34 ± 5.1g	58.9 ± 4.7h	67.8 ± 5.4e	64.55 ± 5.8f	74.61 ± 5.9c	70.03 ± 6.3d	89.5 ± 6.9a	86.5 ± 6.3b
Root length	30.67 ± 2.9h	32.99 ± 2.1g	32.34 ± 2.8g	40.67 ± 3.6e	38.55 ± 3.1f	45.77 ± 3.2c	43.12 ± 3.5d	53.2 ± 4.1a	50.1 ± 4.2b
Pod length	9.23 ± 0.76e	10.21 ± 0.7d	9.97 ± 0.86e	11.4 ± 0.97c	11.3 ± 0.94c	12.76 ± 0.9b	12.87 ± 0.9b	14.7 ± 1.1a	14.5 ± 1.1a
Fresh weight	4.65 ± 0.38f	5.45 ± 0.43e	5.31 ± 0.48e	7.55 ± 0.28c	7.05 ± 0.62d	8.12 ± 0.61b	7.79 ± 0.71c	8.66 ± 0.6a	8.32 ± 0.6b
Dry weight	3.11 ± 0.28f	3.8 ± 0.27d	3.52 ± 0.29e	4.69 ± 0.29c	4.58 ± 0.24c	5.2 ± 0.38b	5.12 ± 0.41b	5.78 ± 0.4a	5.71 ± 0.4a
Leaf area	60.12 ± 4.5i	63.5 ± 4.9g	61.42 ± 4.6h	75.04 ± 6.8e	74.33 ± 6.7f	87.32 ± 7.6c	83.21 ± 7.4d	96.3 ± 8.7a	94.2 ± 8.4b

The standard deviations (SD) are for three replicates, and the different letters denote treatment differences that are statistically significant.

**Table 2:** Impact of CNP treatments on the growth parameters of *Cicer arietinum* seedlings.

Growth parameters	Treatment								
	Control	Ball milled (BM)		Raw fly ash (RFA)		Ball milled-sonicated in nitric oxide (BMN)		Ball milled-sonicated in water (BMW)	
		BM4 (BM4 mg L^−1^)	BM8 (BM8 mg L^−1^)	RFA4 (RFA4 mg L^−1^)	RFA8 (8 mg L^−1^)	BMN4 (4 mg L^−1^)	BMN8 (8 mg L^−1^)	BMW4 (4 mg L^−1^	BM8 (8 mg L^−1^)
Germination (%)	60 ± 5.5h	77.45 ± 6.9f	70.5 ± 6.3g	85.5 ± 7.3d	80.12 ± 7.14e	96.5 ± 7.9b	91.33 ± 7.5c	99.6 ± 8.1a	99.3 ± 8.06a
Shoot length	28.23 ± 1.8h	31.45 ± 2.6g	30.89 ± 2.4g	38.56 ± 2.7e	36.22 ± 2.6f	47.54 ± 3.7c	44.13 ± 3.4d	53.67 ± 4.1a	50.12 ± 4.1b
Root length	19.11 ± 1i	22.09 ± 1.1g	20.87 ± 1.1h	30.98 ± 1.9e	28.65 ± 1.7f	36.89 ± 2.8c	33.12 ± 2.6d	42.56 ± 3.2b	46.77 ± 3.4a
Pod length	9.12 ± 0.86f	10.8 ± 0.96e	10.2 ± 0.98e	12.3 ± 1.1d	11.95 ± 0.98d	14.5 ± 1.4b	14.23 ± 1.1b	15.56 ± 1.5a	15.1 ± 1.5a
Fresh weight	3.92 ± 0.21g	4.35 ± 0.32f	3.96 ± 0.28g	5.57 ± 0.39d	4.72 ± 0.31e	6.13 ± 0.47c	5.97 ± 0.48c	7.98 ± 0.68a	7.32 ± 0.56b
Dry weight	2.54 ± 0.11h	3.23 ± 0.14f	2.92 ± 0.15g	4.12 ± 0.13d	3.54 ± 0.17e	5.1 ± 0.38c	4.92 ± 0.35c	5.66 ± 0.49a	5.34 ± 0.46b
Leaf area	56.45 ± 5.1i	73.66 ± 6.1g	69.99 ± 6.2h	82.12 ± 7.3e	78.05 ± 6.4f	88.5 ± 7.3c	85.34 ± 7.2d	93.56 ± 7.9a	90.21 ± 7.2b

The standard deviations (SD) are for three replicates, and the different letters denote treatment differences that are statistically significant.

**Figure 4:**
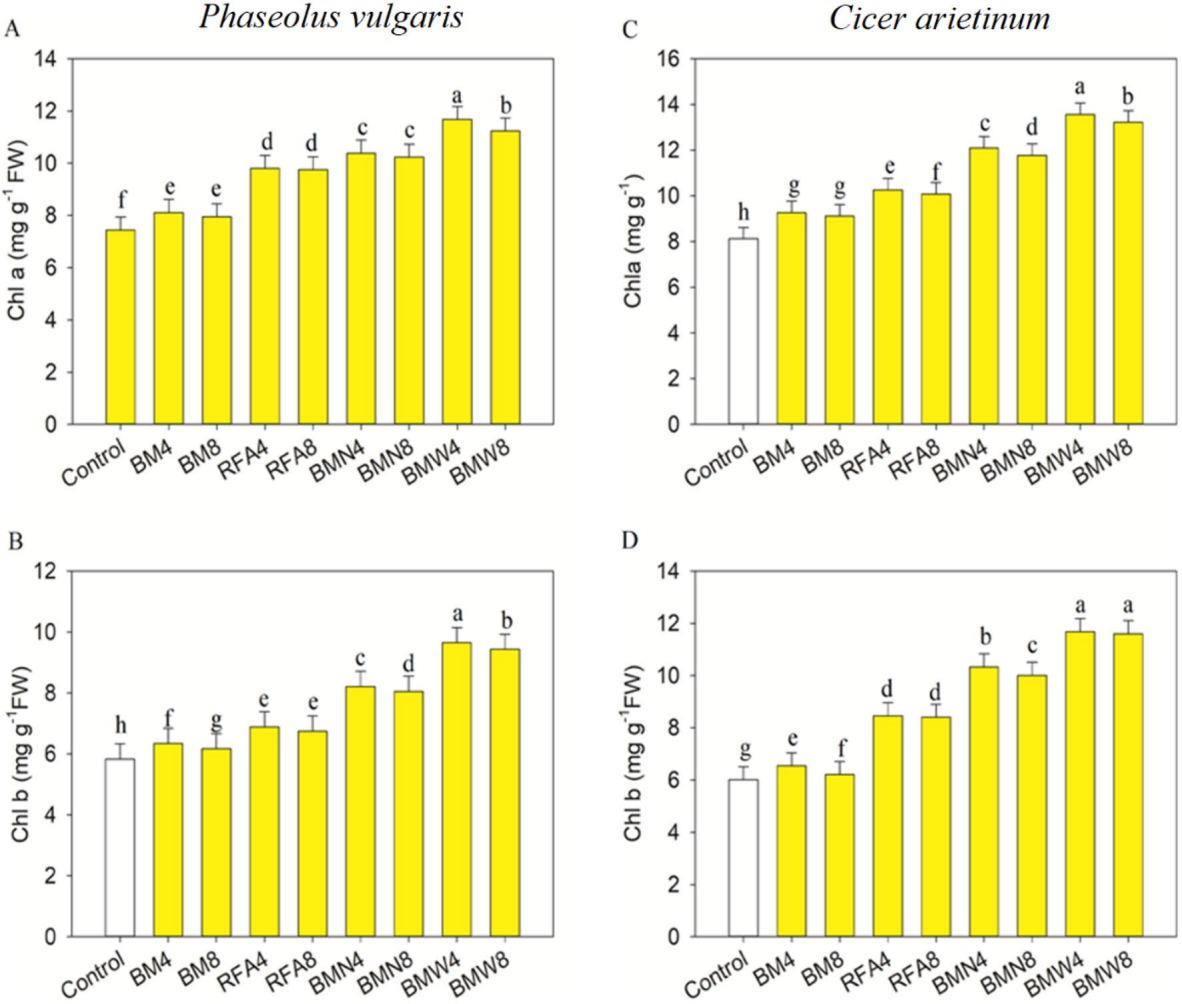
Impact of CNP treatment on the Chl a and Chl b contents of *Phaseolus vulgaris* (**A,B**) and *Cicer arietinum* (**C,D**) seedlings. The bars represent standard deviations (SD) for three replicates, and the different letters denote the treatment differences that are statistically significant.

Data in Fig. 2 clearly show that, the COFA treatments significantly increased (*p* ≤ 0.001) the contents of Chl a, Chl b, carotenoids and total chl pigments in *P. vulgaris,* in comparison with those in the control seedlings. Correspondingly, the COFA treatments led to significant (*p* ≤ 0.001) increases in Chl a, Chl b carotenoids, and total chl with respect to those in the control *C. arietinum* seedlings (Fig. 3, 4). The present data shows that COFA treatment of BMW4 was more effective in increasing all photosynthetic pigments in both *Phaseolus vulgaris* and *Cicer arietinum* seedlings.

**Figure 5:**
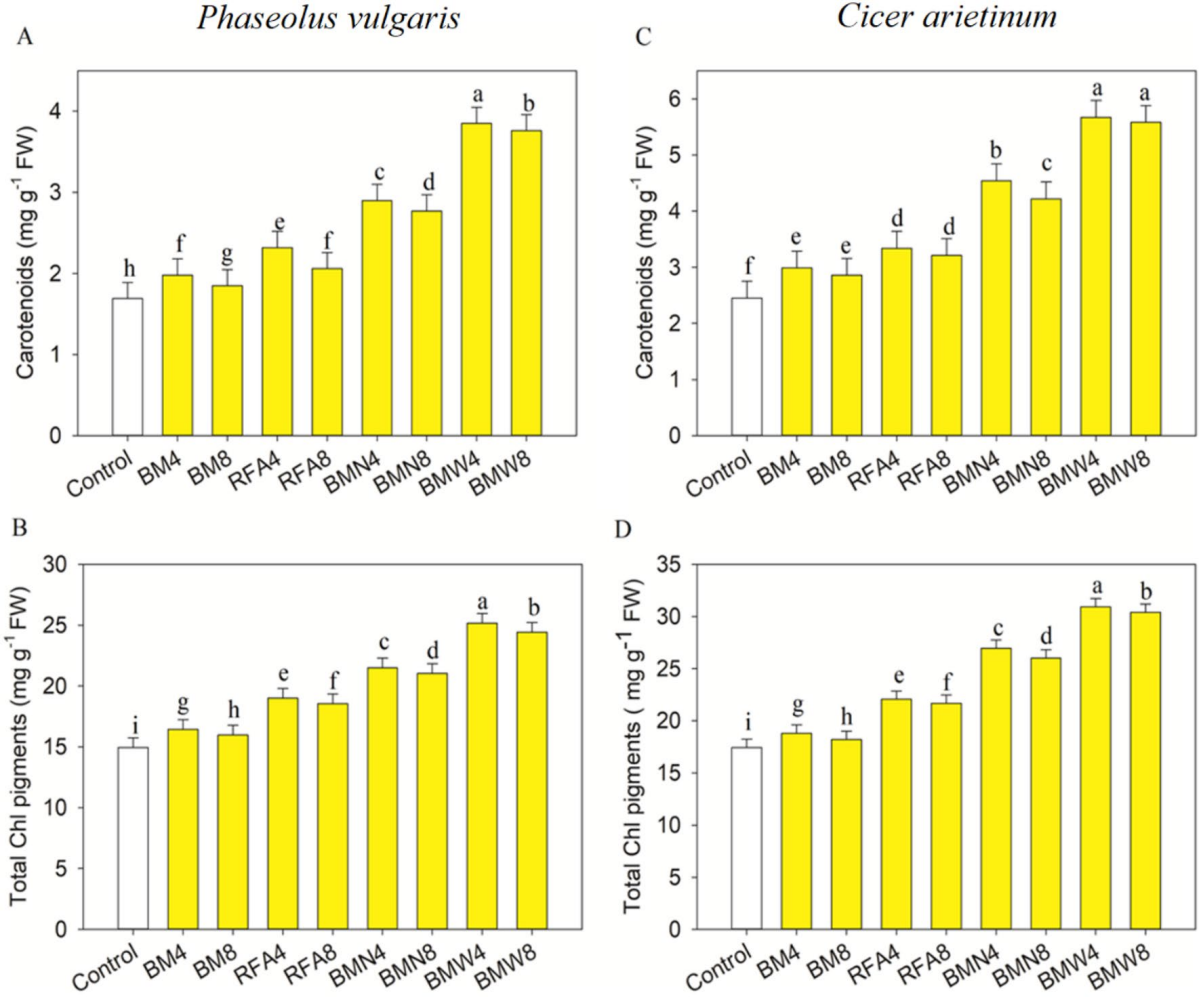
Impact of CNP treatment on the carotenoids and total chl pigments of *Phaseolus vulgaris* (**A,B**) and *Cicer arietinum* (**C,D**) seedlings. The bars represent the standard deviations (SD) of three replicates, and the different letters denote treatment differences that are statistically significant.

**Figure 6:**
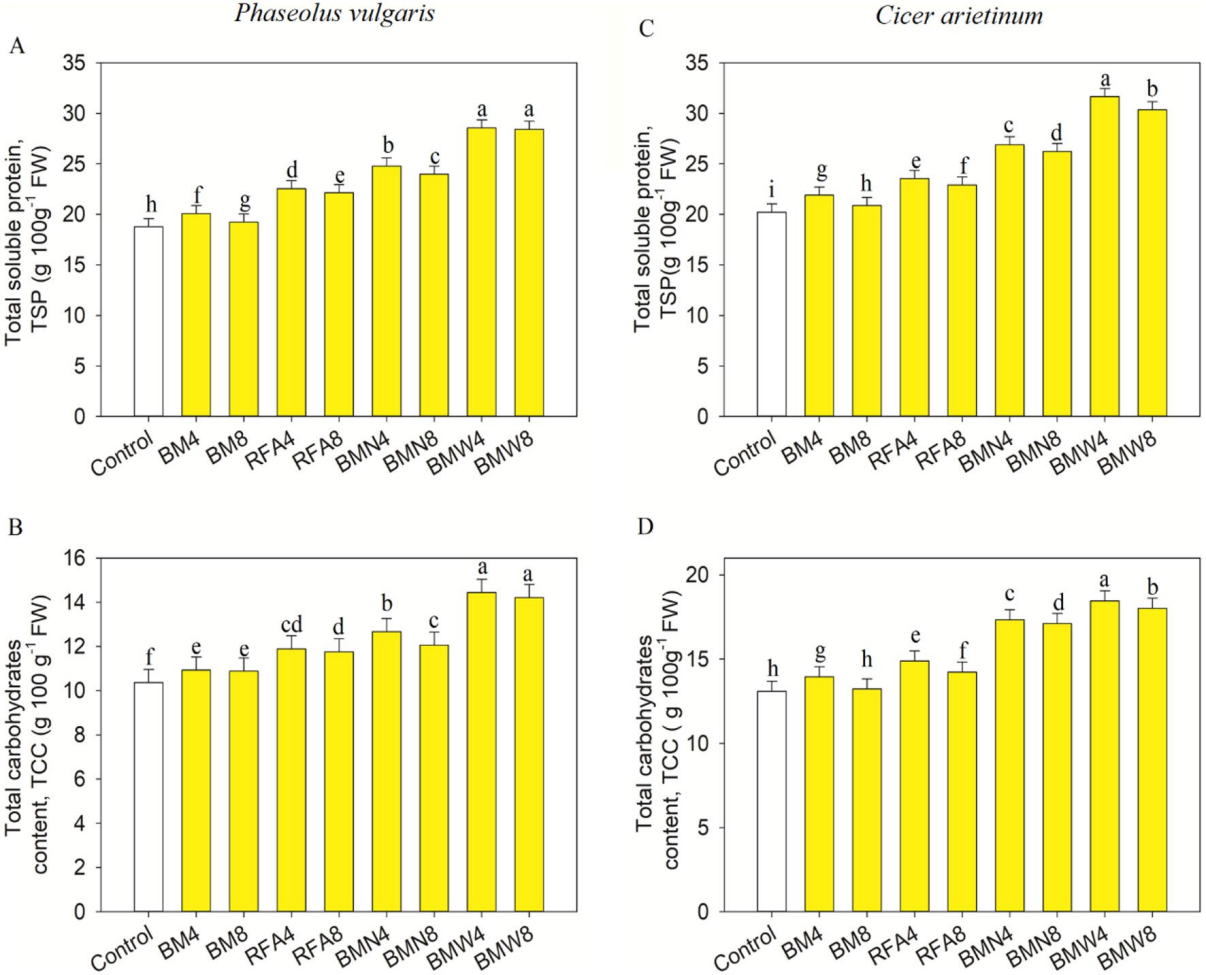
Impact of CNP treatment on the total soluble protein and total carbohydrate contents of *Phaseolus vulgaris* (**A,B**) and *Cicer arietinum* (**C,D**) seedlings. The bars represent the standard deviations (SD) of three replicates, and the different letters denote the treatment differences that are statistically significant.

When COFA was added at doses of BM4, BM8, RFA4, RFA8, BMN4, BMN8, BMW4, and BMW8, the protein contents of *P. vulgaris* rose significantly (*p* ≤ 0.001), when compared to the control. Similarly, the application of COFA increased significantly (*p* ≤ 0.001) the protein content of *C. arietinum* with the BM4, BM8, RFA4, RFA8, BMN4, BMN8, BMW4, and BMW8 doses, respectively, compared to the control (Fig. 5). The COFA treatments led to significant (*p* ≤ 0.001) increases in total carbohydrate content in *P. vulgaris* and *C. arietinum*, with respect to those in control seedlings (Figs. 5, 6). Overall, the results demonstrated that several dosages of COFA treatments induced significant increases in protein and carbohydrate contents in both *P. vulgaris* and *C. arietinum* seedlings, with the maximum concentration obtained at the BMW4 dosage.

## Discussion

Carbon nanoparticles (CNPs) are composed entirely of pure carbon and exhibit exceptional stability, better conductivity, and minimal toxicity when compared to other nanoparticles^24^.

As a result, their application in agriculture to improve the yields of crop plants is critical to sustaining the world's increasing population^25^. The present findings showed that when COFAs interacted with *P. vulgaris* and *C. arietinum* seedlings, they elicited a variety of morpho-physiological changes in agricultural plants, depending on the COFA doses. COFAs were found to have favourable effects on the germination percentages of *P. vulgaris* and *C. arietinum* seeds. Khodakovskaya et al. observed that after 21 days of assessment, the germination rates of seeds and the growth rates of tomato seedlings were significantly improved following exposure to 40 mg L^−1^ CNTs in a MS culture medium^26^.

Plant growth depended on increased CNP penetration^27^. BMW4 (4 mg L^−1^) was shown to be the optimal COFA dosage for plant growth in the current investigation, and all growth parameters were increased. This suggests that responses may vary based on the morphology of the sample used, the dose provided, and the method of application. Our results correspond to those of Li et al.^28^, who found a dose–response relationship between fluorescent carbon dots and the growth parameters of *Vigna radiata*. The study found that up to a concentration of 0.4 mg mL^−1^ for the fluorescent carbon dots, shoot and root lengths first increased but thereafter decreased for greater quantities^28^. The application of CNPs increased the amounts of nitrogen (N) and potassium (K) present in the leaves^29^, which was favourable for seedling growth in *P. vulgaris* and *C. arietinum.* Similar improvements in plant growth resulting from increased N and K concentrations were observed for *N. tabaccum* and *T. aestivum* treated with various concentrations of CNPs, which were much higher than the growth levels achieved with traditional fertilizers^29^.

Chlorophyll pigments play significant roles in photosynthesis as important cofactors of photosystems I and II, as they are responsible for reception of visible light and the photochemical transformations occurring inside the cells^30–33^. Carbon nanoparticles, such as carbon nanotubes and graphene, can easily penetrate epidermal cells and move to cell walls, mitochondria, and chloroplasts via endocytosis^34^. Graphene oxide nanosheets (GNS) are carbon-based synthetic nanomaterials that have been found on cell walls, intercellular gaps, mitochondria, and chloroplasts, indicating that stomata and endocytotic vesicles are involved in GNS uptake^35^. Increases in the number of mitochondrial organelles and accumulations of these organelles adjacent to chloroplasts that were loaded with swollen starch grains were found for the leaves of a pepper plant that had been treated with a graphene nanosheet solution^35^. The plant cells generated sufficient mitochondria to meet the significant amounts of carbohydrates produced upon improving the photosynthetic activity by increasing the chlorophyll content^35^, indicating that CNPs could be important components for improving chlorophyll concentrations. COFA increased the chlorophyll contents of both species in our investigation, which is consistent with earlier findings. For example, when carbon nanomaterials were applied to *S. oleracea*, the quantity and sizes of chloroplasts increased, which led to increases in chlorophyll content and photosynthetic activity^24^. Additionally, increases in the chlorophyll a and b concentrations were observed for a wild carrot (*Daucus carota* L.) crop after application of carbon nanotubes^35^.

Proteins may help plants adapt to oxidative damage by maintaining their structural integrity and changing cell metabolism^36,37^. There have only been a few studies establishing that nanomaterials (NMs) have species-specific and dose-dependent effects on the protein contents of plants^38^. Similar trends for NMs based on metals have demonstrated that protein synthesis was improved in *Spinacia oleracea* L. and *Cyamopsis tetragonoloba* L. plants after treatments with TiO_2_ and ZnO nanoparticles^39,40^. The addition of multiwalled carbon nanotubes increased the protein content of the *Salvia verticillata* plant^41^, which is consistent with our findings.

Carbohydrates are the primary products of photosynthesis, and they are essential for the development of plants since they supply both energy and materials^42^. As a result, increasing the rate of photosynthesis is helpful for accumulating carbohydrate reserves. Carbohydrates were accumulated inside chloroplasts via treatment with GNS, particularly in pepper plants^35^. *P. vulgaris* and *C. arietinum* treated with COFA had higher carbohydrate contents than the control plants, demonstrating that treatment with COFA improved carbohydrate synthesis in plants. This result was consistent with the impact of carbon dots on mung bean carbohydrate content^42^.

## Conclusion

The impacts of COFA on plants are diverse and depend on the morphology of COFA, the dose of the COFA, and the mode of application. The present study concluded that COFA prepared at a concentration of 4 mg L^−1^ via BM sonication in water constituted the best dose for promoting *P. vulgaris* and *C. arietinum* seedling growth. From an agricultural viewpoint, the increases seen for germination rate, leaf area, chlorophyll content, and antioxidant enzyme activities with COFA-treated *P. vulgaris* and *C. arietinum* seedlings are important since they increase the productivities of the plants. Thus, COFA could be a better alternative as a nanofertilizer than conventional fertilizers or manure since it promotes plant growth and improves the plant’s stress tolerance. Additionally, our overall knowledge of the molecular mechanisms underlying the harmful and favourable impacts of COFAs on plants is inadequate. Additionally, future studies should be directed at gaining a better understanding of COFA toxicity toward plants, transportation of COFA inside plant organs, and the impacts of COFA physicochemical characteristics on the root system.

## Data Availability

The datasets used and/or analysed during the current study are available from the corresponding author upon reasonable request.
